# Epidemiological Aspects and Psychological Reactions to COVID-19 of Dental Practitioners in the Northern Italy Districts of Modena and Reggio Emilia

**DOI:** 10.3390/ijerph17103459

**Published:** 2020-05-15

**Authors:** Ugo Consolo, Pierantonio Bellini, Davide Bencivenni, Cristina Iani, Vittorio Checchi

**Affiliations:** 1 Department of Surgery, Medicine, Dentistry and Morphological Sciences with Transplant Surgery, Oncology and Regenerative Medicine Relevance-Unit of Dentistry and Oral-Maxillo-Facial Surgery, University of Modena and Reggio Emilia, 41125 Modena, Italy; ugo.consolo@unimore.it (U.C.); davide.bencivenni@unimore.it (D.B.); vittorio.checchi@unimore.it (V.C.); 2 Department of Surgery, Medicine, Dentistry and Morphological Sciences with Transplant Surgery, Oncology and Regenerative Medicine Relevance-Center for Neuroscience and Neurotechnology, University of Modena and Reggio Emilia, 41125 Modena, Italy; cristina.iani@unimore.it

**Keywords:** COVID-19, Sars-CoV-2, epidemiology, anxiety, dental practice, survey

## Abstract

The outbreak and diffusion of the Severe Acute Respiratory Syndrome-Coronavirus-2 (Sars-CoV-2) and COronaVIrus Disease 19 (COVID-19) have caused an emergency status in the health system, including in the dentistry environment. Italy registered the third highest number of COVID-19 cases in the world and the second highest in Europe. An anonymous online survey composed of 40 questions has been sent to dentists practicing in the area of Modena and Reggio Emilia, one of the areas in Italy most affected by COVID-19. The survey was aimed at highlighting the practical and emotional consequences of COVID-19 emergence on daily clinical practice. Specifically, it assessed dentists’ behavioral responses, emotions and concerns following the Sars-CoV-2 pandemic restrictive measures introduced by the Italian national administrative order of 10 March 2020 (DM-10M20), as well as the dentists’ perception of infection likelihood for themselves and patients. Furthermore, the psychological impact of COVID-19 was assessed by means of the Generalized Anxiety Disorder-7 test (GAD-7), that measures the presence and severity of anxiety symptoms. Using local dental associations (ANDI-Associazione Nazionale Dentisti Italiani, CAO-Commissione Albo Odontoiatri) lists, the survey was sent by email to all dentists in the district of Modena and Reggio Emilia (874 practitioners) and was completed by 356 of them (40%). All dental practitioners closed or reduced their activity to urgent procedures, 38.2% prior to and 61.8% after the DM-10M20. All reported a routinely use of the most common protective personal equipment (PPE), but also admitted that the use of PPE had to be modified during COVID-19 pandemic. A high percentage of patients canceled their previous appointments after the DM-10M20. Almost 85% of the dentists reported being worried of contracting the infection during clinical activity. The results of the GAD-7 (General Anxiety Disorder-7) evaluation showed that 9% of respondents reported a severe anxiety. To conclude, the COVID-19 emergency is having a highly negative impact on the activity of dentists practicing in the area of Modena and Reggio Emilia. All respondents reported practice closure or strong activity reduction. The perception of this negative impact was accompanied by feelings of concern (70.2%), anxiety (46.4%) and fear (42.4%). The majority of them (89.6%) reported concerns about their professional future and the hope for economic measures to help dental practitioners.

## 1. Introduction

From the beginning of 2020, a new pathogen spread from China to Europe and around the globe, and in March 2020, the World Health Organization (WHO) had to officialize a pandemic alert.

This highly infective new virus, named Severe Acute Respiratory Syndrome-Coronavirus-2 (Sars-CoV-2), is a coronavirus responsible of an acute respiratory syndrome, often asymptomatic but potentially lethal [1], named COronaVIrus Disease 19 (COVID-19).

Sars-CoV-2 has an incubation period of two weeks and COVID-19 clinical manifestations mainly include cough, fever and dyspnea [2], but also anosmia, ageusia and, in few cases, diarrhea have been reported [3]. Recently, also cutaneous manifestations have been observed: acral areas of erythema with vesicles or pustules (often after other symptoms) (19%), other vesicular eruptions (9%), urticarial lesions (19%), maculopapular eruptions (47%) and livedo or necrosis (6%) [4]. Airborne and direct contact contamination are the major infection pathways of Sars-CoV-2 [1]. Airborne contamination is due to droplets released through exhalation, cough or sneeze [1]; direct infection instead is due to contact with contaminated surfaces and eye, nose or mouth mucosa [5]. The distance and length of time that particles remain suspended in the air is determined by particle size, settling velocity, relative humidity, and air flow. Droplets that are >5 µm in diameter can spread up to 1 m. The nuclei of the droplets which have a diameter <5 µm, create an aerosol which has a diffusion capacity greater than 1 m [6].

Moreover, it has been reported that virus spread can also happen in absence of clinical symptoms [7,8].

The outbreak and diffusion of Sars-CoV-2 and COVID-19 have caused an emergency status in the worldwide health system. Italy has seen a rapid and massive diffusion of COVID-19 and, as of the 7th of April 2020, Italy registered the third highest number of COVID-19 cases and the second official number of deceased subjects worldwide. The number of Italian cases accounted for 9.47% of total cases worldwide, with 183,957 cases. Of this sample, 94,067 were currently infected (69.37%), 24,391 (17.99%) had recovered, and 17,127 (12.63%) had died [9]. Health care workers are the category with the highest diffusion of the contagion, as the Italian National Institute of Health reports 13,121 cases of infection [9]. Due to droplet production and exposure to saliva and blood, dental practitioners are at high risk of contagion during their routine procedures [1,8,10,11]. SARS-CoV-2 transmission during dental procedures can therefore happen through the inhalation of aerosol/droplets from infected individuals or direct contact with mucous membranes, oral fluids, and contaminated instruments and surfaces [8,9,12].

The aim of this study is to investigate dentist behavior and to analyze their reactions in relation to Sars-CoV-2 pandemic professional restrictive measures due to Italian national administrative order of 10 March 2020 (DM-10M20).

## 2. Materials and Methods 

An online structured survey composed of 40 questions has been sent to dental practitioners in order to investigate dentist behavior and to analyze their reactions in relation to Sars-CoV-2 pandemic restrictive measures introduced by the Italian national administrative order of 10 March 2020 (DM-10M20). The survey focuses mainly on a specific geographical area, the provinces of Modena and Reggio Emilia (the relevant area of our academic institution), one of the areas most involved in the COVID-19 epidemic in Italy. Through the lists of local dental associations (ANDI - Italian Dental Association, CAO – Commissione Albo Odontoiatri) it was sent to all dentists in the area and 40% of them replied.

The survey was created using the free-access Google Forms application and the link to the online survey was sent through an anonymous mailing list to all dentists registered in the Dental Board Commission (CAO) of Modena and Reggio Emilia district. Participants provided their informed consent before completing the survey. Data collection took place in the time period from 2 April to 21 April 2020.

The structured survey was composed of 40 questions, divided into five sections (Table 1).

Section 1 included questions aimed at gathering demographic data (age and gender), and assessing the type of activity and level of experience of the respondents. Section 2 was composed of questions assessing whether practitioners closed their dental practice or reduced their clinical activity following the outbreak of the emergency, whether this occurred before or after the restrictive measures introduced by the Italian government in 10 March 2020 (DM-10M20), which modalities were used to inform patients, and whether patients understood the reasons for the closure/activity reduction. Section 3 was composed of questions investigating the impact of the COVID-19 outbreak on dental practice, which were the most common protective personal equipment (PPE) used before the COVID-19 outbreak and whether habitual PPE had been changed after the outbreak. Section 4 assessed practitioners’ direct or indirect contact with COVID-19, the feelings and emotions experienced while thinking at the COVID-19 outbreak, the dentists’ perception of infection likelihood for themselves and patients. It also assessed the presence of symptoms of anxiety by means of the Generalized Anxiety Disorder 7-item (GAD-7) scale [13], which is commonly used to assess the presence of general anxiety symptoms across various populations and settings. It consists of seven items assessing how often, considering the previous two weeks, individuals have been bothered by COVID-19 related problems: (1) feeling nervous, anxious, or on edge; (2) being able to stop or control worrying; (3) worrying too much about different things; (4) trouble relaxing; (5) being restless; (6) becoming easily annoyed or irritable; (7) feeling afraid as if something awful might happen.

Finally, Section 5 of the survey assessed the practitioners’ main concerns about the professional future, which measures they considered as helpful to support practitioners during and after the emergency, which protective measures they intended to use in the future to prevent the risk of infection for themselves and patients, and whether they believed the emergency situation could lead to improvements.

### Statistical Analysis

Given the nature of our survey we computed descriptive statistics for most of the questions. For each question, we computed the percentage of respondents that gave a particular answer with respect to the number of total responses to the question.

For the questions “How worried are you of contracting COVID-19 during your clinical activity?”, “In your opinion, how likely is it that a patient can contract COVID-19 during a dental service?”, “How much do you think your patients are worried of contracting COVID-19 during a dental service?” and “How worried are you for your professional future?”, response categories were assigned a score ranging from 0 to 4 (0 = “not at all”; 4 = “extremely”).

For the question “Which of the following emotions (fear, anxiety, threat, concern, sadness, anger) do you feel when thinking about COVID-19?” response categories were assigned a score ranging from 0 to 4 (0 = “I do not feel it”, 4 = “I feel it intensely”).

For each of the 7 items of the GAD-7 scale, we assigned the scores 0, 1, 2, and 3 to the response categories “Not at all,” “Several days,” “More than half the days,” and “Nearly every day”, respectively. The scores for each item were then summed to obtain a total score ranging from 0 to 21. Scores from 0 to 4, from 5 to 9, from 10 to 14 and from 15 to 21 are indicative of minimal, mild, moderate and severe anxiety, respectively.

We computed the Pearson correlation coefficient to investigate the association between general anxiety level, as indexed by the GAD-7 general score, level of concern for the professional future, level of concern of contracting the COVID-19, perceived patient’s likelihood of contracting the infection, and the level of concern of contracting the infection attributed to the patient. We also investigated the association between the impact of COVID-19 on dental practice and level of concern about the professional future. Furthermore, to assess potential differences between age groups, we submitted the mean scores obtained in the questions reported above and the GAD-7 score to a one-way Analysis of Variance (ANOVA) with age group (<35 years, 35 and 55 years, and >55 years) as a between-participants factor. Statistical analyses were performed using the SPSS version 26.0 statistical software.

## 3. Results

The survey was sent to 874 practitioners and 356 of them completed it. With this sample size, the margin error at a 95 level of confidence is lower than 5%.

Of the respondents, 60.4% were male and 39.6% were female. The majority of participants were aged between 35 and 55 (48.6%); 34.8% were over 55 years old, while only 16.6% of them were under 35 years old. Consequently, most had been working for more than 15 years (61.2%), 28.4% had been working for 6–14 years, while 10.4% had been working for less than 5 years. A large number of dentists (226; 63.5%) reported working 30–40 h or more per week, while the remaining 130 (35.5%) reported working less than 30 h per week. The majority of the compilers were practice owners (64.3%), while the others were private (34.6%) or public (1.1%) structures employees (Table 2).

All of the respondents closed or highly reduced their activity to urgent procedures, 38.2% before and 61.8% after the DM-10M20. Patients were contacted mainly by phone (95.8%), only 4.2% through social channels or websites. Most of them understood the reasons for the closure of dental practices or for the reduction in clinical activity (93%). A high percentage of patients (92.7%) canceled their previously-taken appointments after the DM-10M20. A large number of dentists (342, 96.1%) guaranteed telephone availability for dental emergencies. Almost the totality of compilers (321, 90.2%) reported the willingness to personally take care of emergency situations. When an emergency occurred, 45% of respondents took care of it alone, and 55% of them were helped by an assistant.

Approximately 70% of practice owners reported an average number of 6 to 15 patients a day before the pandemic, that shifted to 0 to 5 a week in 90% of the sample.

Each practitioner asserted a routinely use of the most common protective personal equipment (PPE), such as gloves, masks, disposable gowns and protective glasses before the Sars-CoV-2 pandemic (Table 3).

However, they also admitted that they had to increase the use of PPE or to modify kinds of PPE during the COVID-19 pandemic (77%), or that they were still awaiting directives to do so (12.9%). Only 10% have not changed their PPE, probably because they were already applying maximal PPE before the pandemic. Since the beginning of coronavirus pandemic, 86% of the respondents reported difficulties in finding PPE, and 57.9% reported problems in the delivery time of dental materials. Most of the interviewees (279, 78.4%) report having held information sessions dedicated to the staff on the correct use of PPE, 13.2% did not, but 8.4% said that they will soon.

Fortunately, only four (1.1%) respondents contracted COVID-19, while 68.6% knew at least one person who has been infected. In total, 20.8% did not know anyone who has contracted the disease.

For 74.4% of the respondents, COVID-19 was having a highly negative impact on their professional activity (Mean (M) = 3.7, Standard Deviation (SD) = 0.7) and the majority of them (89.6%) was quite concerned about their professional future (M = 2.7, SD = 1.02), mostly due to the uncertainty about the end of the emergency situation. The level of concern about the future was positively correlated to the reported level of negative impact (Pearson’s correlation index: r = 0.17, *p* < 0.001).

Dentists reported being quite concerned of contracting COVID-19 during their clinical activity (M = 2.52, SD = 1.02). More precisely, 20.2% were extremely concerned, 29.2% were very concerned and 35.7% quite concerned. Only 12.6% were little concerned while 2.2% were not concerned at all. 38.2% of them believed patients’ concern of contracting the infection during a dental visit was quite high (M = 1.73, SD = 1.06), even though they overall considered the patient’s likelihood of infection as low (M = 1.25, SD = 1.11) (Table 4).

When thinking about COVID-19, only 4.2% of the respondents reported to experience fear intensely, while the majority reported to feel lightly (41%) or moderately (23.9%) scared. Only 6.2% reported to experience anxiety intensely, while the majority reported to feel lightly (37.4%) or moderately anxious (23.6%). Only 16% reported to experience concern intensely, while the majority reported levels of concern ranging from light (26.4%) to moderate (29.8). Only 12.6% of respondents felt intensely sad, while 25.3% did not experience sadness at all. Anger was experienced in an intense way by only 9.3% of respondents, while 44.1% of respondents did not experience anger at all. Overall, these results indicate that thinking about COVID-19 mostly caused concern (M = 2.23, SD = 1.11) (Table 5).

The mean GAD-7 score was 6.56 (SD = 4.48) indicating an overall mild level of general anxiety. More precisely, 42.7% of the respondents showed minimal anxiety (score 0–4), 33.3% showed mild anxiety (score 5–10), 15.2% showed moderate anxiety (score 10–14), while 8.7% showed a score indicative of a severe level of anxiety (score 15–21). The GAD-7 score was positively correlated to the level of concern about the professional future (r (356) = 0.32, *p* < 0.001), the level of concern of contracting the COVID-19 shown by the dentists (r (356) = 0.26, *p* < 0.001), the perceived patient’s likelihood of contracting the infection (r(356) = 0.23, *p* < 0.001), and to the level of concern attributed to patients (r(356) = 0.28, *p* < 0.001).

The one-way ANOVA showed a main effect of age group for perceived patient’s likelihood of contracting the infection (F_2,353_-Statistic = 1157, *p* < 0.001), and reported levels of concern about the professional future (F_2,353_ = 4.18, *p* < 0.05). Post-hoc comparisons showed that perceived patient’s likelihood of contracting the infection was lower (M = 0.88, SD = 0.89) for dentists older than 55 years of age as compared to the other two age groups (<35 years: M = 1.56, SD = 1.07; 35–55 years: M = 1.40, SD = 1.19). Dentists aged between 35 and 55 years were those more concerned about their professional future (M = 2.86, SD = 0.97) compared to the other two age groups (<35 years: M= 2.58, SD = 1.12; >55 years: M = 2.54, SD = 1.02).

To the question “During clinical activity, which measures do you use to prevent COVID-19 infection?”, dentists replied highlighting a good knowledge of what is reported in the most recent indications from the literature. This question could be answered by placing multiple preferences: the highest frequency of answers concerned “Reduction of number of patients in the waiting room” (87.1%) and “Telephone screening/anamnesis to exclude COVID-19 related symptoms” (86.5%). Less frequently, “Environment aeration” (77.5%), “Use of PPE” (73.3%) or “Disinfectant agents and surgical mask supply to all patients while waiting in waiting room” (68.8%) were indicated. Other indications, provided by medical organizations and media—“Environment sanitation” and “Telephone screening/anamnesis to identify possible critical cases”—received 65.5% and 43.5%, respectively. The answer “Body temperature measurement” received the lowest frequency of preferences (21.3%).

The same question, repeated at the end of the questionnaire with reference to future behaviors, highlighted percentage variations: “Reduction of number of patients in the waiting room” (84.8%), “Use of PPE” (82.6%), “Telephone screening/anamnesis to identify possible critical cases” (78.4%), “Environment aeration” (75.3%), “Environment sanitation” (74.7%), “Disinfectant agents and surgical mask supply to all patients while waiting in waiting room” (66%) and “Body temperature measurement” (35.7%).

To the question “Which aids do you think could help dental professionals during COVID-19 pandemic?”, for which two preferences could be expressed, the dentists replied indicating “Economic relieves from Italian government” (65.7%), “Social security institutions support and subsidy” (44.1%),” Economic relieves from dental associations” (32.1%) and “Improvement of communication with patients” (8.1%).

The answers to the successive question, which analyzes the category aid measures to be adopted after the emergency, maintained almost the same order of frequency in the answers. There was a decrease in the percentage for “Social security institutions support and subsidy” and 9.6% for “Bank account support”, which was not represented in the answers to the previous question. In descending order, the percentages were: “Economic relieves from Italian government” (73.9%), “Economic relieves from dental associations” (31.2%), “Social security institutions support and subsidy” (26.1%), “Improvement of communication with patients” (16%) and “Bank account support” (9.6%). Greater importance was given to communication campaigns with patients.

The last question asked “Which improvements do you think can result from the COVID-19 emergency?” and multiple answers could be indicated. Most of the interviewees considered “Prevention procedures standardization” very important (66.9%) and a high percentage answered that there will be a “Professional rhythm slow down” (36.8%) and “Improvement of communication with patients” (23%). Lower preferences resulted for “No improvements” (19.9%) and “Stabilization of relationship with dental associations” (16.9%). Dentists considered the “Reduction of dental practices competition” irrelevant, which received the smallest number of indicated preferences (5.1%).

## 4. Discussion

Since the Sars-CoV-2 pandemic, other surveys have been proposed by other international institutions, aimed at measuring the impact of this turmoil on dental professionals. One inquiry was performed in Israel [14], a nation where the impact of the COVID-19 has been much more contained than in Italy. Another survey, form Saudi Arabia [15], had a more global reach: 650 dentists spread out in many countries, mostly in Pakistan, India and Malaysia, where the dental setting might differ from Western standards and where the majority of the colleagues are employed in public settings. Our survey is exclusively focused on a specific geographical area, the province of Modena and Reggio Emilia (the pertinent area of our academic institution) in northern Italy, one of the most involved areas in the COVID-19 outbreak in Italy and, perhaps, in Europe. It reached out to 874 dentists, through the lists of the local dental associations (ANDI, CAO), and 40% of them responded. The questions on the survey were developed after reviewing pertinent literature and international guidelines [10,14,15,16]. The questionnaire was designed in the Italian language and comprised of questions pertaining to socio-demographic characteristics, dentists’ attitudes and perceptions toward COVID-19 and infection control in dental clinics. Moreover, the investigation was also focused on the psychological impact and changes on the everyday dental practice.

The survey was a structured multiple-choice questionnaire divided into four sections.

Section 1 section centered on practice and owner socio-demographical characterization: age, gender, years of service, number of operative units, number of dental assistants and collaborators. Among respondents, the majority were male (60.4%) and private practice owners (64.3%), working on average in 2–3-unit offices, whilst the other part were private or public structures employees. Almost half of the sample was aged between 35 and 55. Young dentists, aged 35 years old or less, accounted for 16.6%.

Section 2 is focused on the actual and real impact of the COVID-19 outbreak on dental practice nowadays: the totality (100%) of owners closed their dental offices (38.2% before the DM-10M20 and 61.8% after), assuring telephone availability in 96.1% of cases. It was not only the colleagues that were afraid of the situation, but also patients were probably aware of the risks in the dental office, since 92.7% reported cancellation directly from patients, just before the DM-10M20. As a matter of fact, three-fourths of the interviewees reported that there has been an extremely negative impact on their practices.

Section 3 is about the adaptive behavior to the pandemic outbreak and risk perception. This has been evaluated through the need for PPE implementation, the need for informative sessions about their correct utilization and through a Generalized Anxiety Disorder-7 test (GAD-7). SARS-CoV-2 has been demonstrated to remain aerosolized for 3 h after contamination and on plastics and stainless steel for up to 72 h [17]. This makes the dental community a relatively high-risk population [1]. There are practical guidelines recommended for dentists and dental staff by the Centers for Disease Control and Prevention (CDC), the American Dental Association (ADA) and the World Health Organization to control the spread of COVID-19 [18,19,20]. Like with other contagious infections, these recommendations include personal protective equipment, hand washing, detailed patient evaluation, rubber dam isolation, anti-retraction handpiece, mouth rinsing before dental procedures, and disinfection of the clinic.

In our survey, the vast majority performed a telephonic triage the day before the appointment, along with a full-body protection during the operative procedure. The necessity to reduce the number of incoming patients in the waiting room was held important by 87.1% of the colleagues. The way patients are received in the dental office has been modified as well, since 68.8% is providing patients with surgical mask and hand sanitizer upon arrival. Surprisingly, only a small minority is considering the body temperature check upon entrance as a valid method for critical case detection notwithstanding the low cost and the good reliability of this procedure. It must be remembered that the current approach to COVID-19 is to control the source of infection; use infection prevention and control measures to lower the risk of transmission and provide early diagnosis, isolation, and supportive care for affected patients. Based on relevant guidelines and research, dentists should take strict personal protection measures and avoid or minimize operations that may produce droplets or aerosols [21].

Only 1.1% of the practitioners referred positivity to COVID-19, whereas 68.6% has at least one patient/collaborator/friend that tested positive, so this pandemic is definitely a reality in our settings.

It is of interest to note that the majority of practitioners fear infection, but only a minority group is concerned about the possibility that their patients might acquire the infection. The fear of contracting COVID-19 from a patient is strongly associated with elevated psychological distress. Similar results are reported in a survey conducted in Israel: dentists’ responses to prevention measures seem better for personal protective equipment, disinfection and sanitation procedures than for measures applied to patients [14]. This could mean that the majority of the interviewees are more concerned about protecting themselves than their patients.

Measuring anxiety by the means of self-report questionnaires is useful [22] and has been already performed among dental practitioners and patients [23]. In this survey, fear, anxiety, concern, sadness and anger are commonly reported, but fortunately only a minority group reported intense feelings of anger (9.3%) and, as resulting from the GAD-7 scale, inability to manage anger and anxiety (10.3%). Overall, only 8.7% of the respondents showed a score to the GAD-7 scale indicative of a severe level of anxiety. The overall level of general anxiety can be considered as mild (mean GAD-7 score was 6.56, SD = 4.48). These data are consistent with those reported by another survey in Israel in which elevated psychological distress was found in 11.5% of the sample [14].

What is most expected is the receipt of prompt support from both the national government and the physicians’ social security institution (ENPAM–Ente Nazionale di Previdenza ed Assistenza). Informative communication for patients is believed to be important to let them know how problems in dental offices are being ameliorated.

Section 4 of the essay is about the perception of our professional future. A pandemic often brings economic recession, and this is what happened during the first quarter of 2020. This pandemic will have an impact on every aspect of our global economy. Some analysts have predicted that—owing to the measures enacted to stop the spread of this pandemic, such as large-scale quarantines, travel restrictions, and social-distancing measures—there will be a sharp decrease in consumer and business spending capacity until the end of 2020 and part of 2021 [24]. This will ultimately lead to a global recession. As health-care professionals, dentists have responsibilities and should explore long-term measures to avoid recrudescence and future outbreaks. This situation will be challenging for medicine and dentistry, and the financial impact on dental practices will be experienced in both the short- and long-term.

It is important to note that the vast majority of the respondents reported apprehension about the professional future. What is alarming the most is the inability to prevent the end of the pandemic, followed by the impaired economy that might affect future patient turnover and the capability to pay for the dental practice expenses. Moreover, one third of the interviewees expressed concern about the need to buy further devices and to adequate to new clinical protocols to counteract the spreading of SARS-CoV-2. This will probably result in some physicians and dentists going out of business, especially the oldest (and more experienced) ones, and might also prevent new generation dental practitioners to get into business. Dentists aged between 35 and 55 years were the most concerned about their professional future.

What colleagues expect as a support to adequately face their professional future is the receipt of benefits from the Italian government and social security institutions, as well as from Italian Dental Associations (CAO, ANDI). The government will pay laid off staff for a period; however, this is only a portion of most doctors’ overall costs. The dental private sector is already facing a financial crisis and this is expected to worsen, primarily due to the need of providing a better and safer working environment to our patients, staff, and ourselves. This will potentially increase business overheads and reduce profit margins even further. 

Alternatively, professionals could start to conceptualize new paradigms and a new vision about their profession. Telehealth has become an essential tool for providing care to patients [10]. It is already allowing physicians to connect with patients sparing costs and time. Its use will definitely exponentially increase over time and it might become an interesting tool for dental care providers as well. Dentists and oral surgeons could integrate it into their clinical practice. Potential uses include preoperative and postoperative visits as well as follow-up controls, thus reducing patient coming and going in our offices. This innovation has actually received good acceptance from patients, government and health-care providers in the U.S. and can represent a new business opportunity for our colleagues [25].

The general feeling among our respondents is such that their profession will change for a long time: harsh preventive measures are felt to be necessary in the near future, such as access limitation to the waiting room, more adequate protection devices, decontamination of the working environment, but still, the body temperature check, upon patient arrival, is considered necessary only by a minority of colleagues. The answers collected by our survey are quite consistent with general recommendations provided to dentists and to other health-care providers world-wide [10,16,18,19,20,21]. Patients should be asked about their health status and any history of recent contact or travel; patients and their accompanying persons should be provided with medical masks upon entry to the clinic. Patients with body temperature >37° should be registered and referred to designated family doctors. If a patient has been to any epidemic regions within the past 14 days, quarantining for at least 14 days is recommended.

At last, our survey is focused on the perception of the professional improvement: what could positively change as a consequence of the pandemic. Only less than 20% believe that no improvements will occur. The majority believes that some ameliorations will arise: new standardized preventive procedures, a slow-down in the working-schedule, improvements in communicating with patients and even a diminished competition between dental practices. It is possible to foresee a better awareness about new and strict preventive protocols among dentists as a positive achievement for the category. The AIDS pandemic resulted in acceptance of solutions that revolutionized the standard of care throughout medicine. Prior to HIV/AIDS, dentists did not commonly wear gloves, masks or eye protection [26,27]. In the late 1980s and early 1990s, in an attempt to protect health care workers, CDC proposed guidelines to reduce exposure to blood-borne pathogens such as HIV and hepatitis B [28]. Dentistry curbed this change at every step but these standards of protections are widely accepted and used nowadays. What will come of this pandemic? Commercial air purifiers and air exchange devices are also being explored for dental settings [29]. Creating negative pressure operatories may seem a drastic and expensive approach now, but it may become a normal standard a few years from now.

Despite the findings discussed above, it is important to stress that this survey had a major limitation, due to the fact that our investigation regarded a relatively small area in north Italy—the province of Modena and Reggio Emilia—and this prevents us being able to generalize our results.

## 5. Conclusions

The COVID-19-related emergency condition is having a highly negative impact on dental practices in the area of Modena and Reggio Emilia—the area of our academic institution. All of the dentists that completed the survey reported practice closure or reduction, a high level of concern about the professional future and the hope of economic funding for all dental practitioners. Concerns related to professional activity were accompanied by severe anxiety levels for a small percentage of respondents. This essay must be contextualized with the geographical area, northern Italy—one of the most involved in terms of pandemic—and was delivered during the most critical period of the pandemic. This might have brought a sort of bias in the psychological profiling: Probably more pessimistic answers could be anticipated. Importantly, some improvements are expected to be derived from the actual emergency situation, such as the adoption of standardized preventive procedures, a slow-down in working-schedule, and even diminished competition between dental practices.

## Figures and Tables

**Table 1:** Survey composed of 40 questions that has been sent in order to investigate dentist behavior and to analyze their reactions in relation to Severe Acute Respiratory Syndrome-Coronavirus-2 (Sars-CoV-2) pandemic restrictive measures.

Questions	Answer(s)			
1. Do you want to participate in this survey?	Yes			
	No			
2. Age	<35 year			
	35–55 year			
	>55 year			
3. Sex	Male			
	Female			
4. Dental practice in: *[One answer allowed]*	Modena			
	Reggio Emilia			
	Other (specify)			
5. Professional experience *[One answer allowed]*	0–5 years			
	6–10 years			
	11–15 years			
	>15 years			
6. Professional setting *[One answer allowed]*	Owner of private practice			
	Partner or employed in private practice			
	Employed in public structure			
7. Number of dental chairs *[One answer allowed]*	One			
	Two/Three			
	>Three			
8. Number of dental assistants and/or secretaries *[One answer allowed]*	One			
	Two/Three			
	>Three			
9. Number of partners or employees *[One answer allowed]*	None One >One			
10. Weekly average working time *[One answer allowed]*	<20 h			
	20–30 h			
	30–40 h			
	>40 h			
11. Average number of patients treated daily before Italian national administrative order of 10 March 2020 (DM-10M20)				
12. Due to COronaVIrus Disease 19 (COVID-19), was the practice closed/reduced to urgent procedures only? *[One answer allowed]*	Yes			
	No			
13. When was the practice close or clinical activity reduced to urgent procedures only? *[One answer allowed]*	Before DM-10M20			
	After DM-10M20			
14. A telephonic availability was guaranteed for dental emergencies? *[One answer allowed]*	Yes			
	No			
15. In case of dental emergencies, did you personally take care of them? *[One answer allowed]*	Yes			
	No			
16. In case of dental emergencies, were the dental assistant(s) present? *[One answer allowed]*	Yes			
	No			
17. Did patients understand motivations for practice closure/clinical activity reduction? *[One answer allowed]*	Yes			
	No			
18. How were patients notified of practice closure/clinical activity reduction? *[Multiple answers allowed]*	By telephone			
	By email			
	By web site			
	By social networks (Facebook, Instagram, etc.)			
19. Average number of patients treated daily after DM-10M20				
20. Did patients cancel their previously-taken appointments after DM-10M20? *[One answer allowed]*	Yes			
	No			
21. Did COVID-19 pandemic condition negatively your professional activity? *[One answer allowed]*	Not at all			
	Little			
	Quite			
	A lot			
	Extremely			
22. Which Personal Protective Equipment (PPE) were used usually before COVID-19? *[One answer allowed]*	Gloves and surgical masks			
	Gloves, surgical masks and disposable isolation gowns			
	Gloves, surgical masks, disposable isolation gowns, disposable protective cap and glasses/face shield			
	Others (Specify)			
23. Since DM-10M20, did you modify the choice of PPE? *[One answer allowed]*	Yes			
	No			
	No, I’m waiting government guidelines			
24. Since DM-10M20, did you hold informative sessions dedicated to coworkers and employees on the correct use of PPE? *[One answer allowed]*	Yes			
	No			
	No, but I will do it			
25. Dental associations had been useful in giving instructions about PPE? *[One answer allowed]*	Yes			
	No			
	I don’t know			
26. During clinical activity, which measures do you use to prevent COVID-19 infection? *[Multiple answers allowed]*	Telephone screening/anamnesis to exclude COVID-19 related symptoms			
	Telephone screening/anamnesis to identify possible critical cases			
	Reduction of number of patients in the waiting room			
	Body temperature measurement			
	Environment aeration			
	Environment sanitation			
	Disinfectant agents and surgical mask supply to all patients while waiting in waiting room			
	Use of PPE (Respirator masks, disposable gowns, double layered gloves, etc.)			
	Other (specify)			
27. Since the beginning of the pandemic, did you have difficulties in finding PPE? *[One answer allowed]*	Yes			
	No			
	I don’t know			
28. Since the beginning of the pandemic, have you noticed delays in the delivery timing of dental materials? *[One answer allowed]*	Yes			
	No			
	I don’t know			
29. Do you know someone who contracted COVID-19? *[Multiple answers allowed]*	Me			
	One or more relatives			
	On or more employees			
	One or more patients			
	One or more acquaintances			
	No			
30. How worried are you of contracting COVID-19 during your clinical activity? *[One answer allowed]*	Not at all			
	Little			
	Quite			
	A lot			
	Extremely			
31. In your opinion, how likely is it that a patient can contract COVID-19 during a dental service? *[One answer allowed]*	Not at all			
	Little			
	Quite			
	A lot			
	Extremely			
32. How much do you think your patients are worried of contracting COVID-19 during a dental service? *[One answer allowed]*	Not at all			
	Little			
	Quite			
	A lot			
	Extremely			
33. Which of the following emotions do you feel when thinking about COVID-19?	Fear			
	Anxiety			
	Concern			
	Sadness			
	Anger			
34. How frequently one of these issues bothered you in the past two weeks?				
	Not at all	Several days	More than half the days	Nearly every day
Being more nervous and/or anxious				
Being unable to stop to worrying				
Being too much worried for various things				
Having difficulties in relaxing				
Being agitated and unable to stay still				
Getting easily irritated				
Having fear that something terrible could happen				
35. How worried are you for your professional future? *[One answer allowed]*	Not at all			
	Little			
	Quite			
	A lot			
	Extremely			
36. What worries you the most? *[Multiple answers allowed]*	I don’t know when this emergency situation will end			
	Patients will have less money to spend			
	The crisis of dental environments will get worse			
	The need of new procedures and new devices for safety and infection prevention			
	The chance of losing my job or having to fire my employees			
37. Which aids do you think could help dental professionals during COVID-19 pandemic? *[Multiple answers allowed]*	Economic relieves from Italian government			
	Improvement of communication with patients			
	Economic relieves from dental associations			
	Bank account support			
	Social security institutions support and subsidy			
38. Which aids do you think could help dental professionals after COVID-19 pandemic? *[Multiple answers allowed]*	Economic relieves from Italian government			
	Improvement of communication with patients			
	Economic relieves from dental associations			
	Bank account support			
	Social security institutions support and subsidy			
39. During clinical activity, which measures will you use to prevent COVID-19 infection? *[Multiple answers allowed]*	Telephone screening/anamnesis to identify possible critical cases			
	Reduction of number of patients in the waiting room			
	Body temperature measurement			
	Environment aeration			
	Environment sanitation			
	Disinfectant agents and surgical mask supply to all patients while waiting in waiting room			
	Use of PPE (Respirator masks, disposable gowns, double layered gloves, etc.)			
	Other (specify)			
	Prevention procedures standardization			
40. Which improvements do you think can result from the COVID-19 emergency? *[Multiple answers allowed]*	Reduction of dental practices competition			
	Improvement of communication with patients			
	Professional rhythm slowdown			
	Stabilization of relationship with dental associations			
	No improvements			

**Table 2:** Demographic information of dental practitioners (*n* = 356).

Demographics		Number (%)
Gender	Male	215 (60.4)
	Female	141 (39.6)
Age (years)	Less than 35	59 (16.6)
	35–55	173 (48.6)
	Above 55	124 (34.8)
Type of activity	Practice owner	229 (64.3)
	Private practice employee	123 (34.6)
	Public structure employee	4 (1.1)
Work experience (years)	0–5	37 (10.4)
	6–10	43 (12.1)
	11–15	58 (16.3)
	More than 15	218 (61.2)
Numbers of hours worked per week	Less than 20	37 (10.4)
	20–30	78 (21.9)
	30–40	93 (26.1)
	More than 40	148 (41.6)

**Table 3:** Combination of personal protective equipment (PPE) used before COVID-19 (*n* = 356).

PPE	Number (%)
Only gloves and mask	123 (34.6)
Gloves, mask and disposable isolation gown	44 (12.4)
Gloves, mask, disposable isolation gown, disposable protective cap and protective glasses/face shields	151 (42.4)
Other	38 (10.7)

**Table 4:** Dentists’ concern of contracting COVID-19, perception of the infection likelihood for patients and level of concern attributed to patients.

Question (Score 0–4)	Not at all 0 *N* (%)	Little 1 *N* (%)	Quite 2 *N* (%)	A lot 3 *N* (%)	Extremely 4 *N* (%)	Mean (SD)
How worried are you of contracting COVID-19 during your clinical practice?	8 (2.2)	45 (12.6)	127 (35.7)	104 (29.2)	72 (20.2)	2.52 (1.02)
In your opinion, how likely is it that a patient can contract COVID-19 during a dental service?	98 (27.5)	140 (39.3)	69 (19.4)	30 (8.4)	19 (5.3)	1.25 (1.11)
How much do you think patients are worried of contracting COVID-19 during a dental procedure?	57 (16.0)	78 (21.9)	136 (38.2)	73 (20.5)	12 (3.4)	1.73 (1.06)

**Table 5:** Which of the following emotions do you feel when thinking about COVID-19? (n = 356).

Emotions (Score 0–4)	I Do Not Feel It 0 *N* (%)	Lightly 1 *N* (%)	Moderately 2 *N* (%)	Quite Intensely 3 *N* (%)	Intensely 4 *N* (%)	Mean (SD)
Fear	59 (16.6)	146 (41.0)	85 (23.9)	51 (14.3)	15 (4.2)	1.49 (1.06)
Anxiety	58 (16.3)	133 (37.4)	84 (23.6)	59 (16.6)	22 (6.2)	1.59 (1.13)
Concern	12 (3.4)	94 (26.4)	106 (29.8)	87 (24.4)	57 (16.0)	2.23 (1.11)
Sadness	90 (25.3)	70 (19.7)	85 (23.9)	66 (18.5)	45 (12.6)	1.74 (1.35)
Anger	157 (44.1)	83 (23.3)	43 (12.1)	40 (11.2)	33 (9.3)	1.18 (1.35)
